# Associated features in females with an *FMR1* premutation

**DOI:** 10.1186/1866-1955-6-30

**Published:** 2014-07-30

**Authors:** Anne C Wheeler, Donald B Bailey Jr, Elizabeth Berry-Kravis, Jan Greenberg, Molly Losh, Marsha Mailick, Montserrat Milà, John M Olichney, Laia Rodriguez-Revenga, Stephanie Sherman, Leann Smith, Scott Summers, Jin-Chen Yang, Randi Hagerman

**Affiliations:** 1 RTI International, 3040 Cornwallis Road, Research Triangle Park, NC 27709, USA; 2 Rush Medical Center, 1735 West Harrison Street Suite 718, Chicago, IL 60612, USA; 3 Waisman Center, University of Wisconsin, 1500 Highland Avenue, Madison, WI 53705, USA; 4 Northwestern University, 2240 Campus Drive, Evanston, IL 60208-3507, USA; 5 Biochemistry and Molecular Genetics Department, Hospital Clinic, Villarroel 170, 08036 Barcelona, Spain; 6 Center for Mind and Brain, University of California-Davis, 1 Shields Avenue, Davis, CA 95616, USA; 7 Emory University, 615 Michael Street, Atlanta, GA 30322, USA; 8 MIND Institute, University of California Davis, 2825 50th Street, Sacramento, CA 95817, USA; 9 Carolina Institute for Developmental Disabilities, University of North Carolina at Chapel Hill, Chapel Hill, NC 27599, USA

**Keywords:** fragile X, *FMR1* premutation, health risks

## Abstract

Changes in the fragile X mental retardation 1 gene (*FMR1*) have been associated with specific phenotypes, most specifically those of fragile X syndrome (FXS), fragile X tremor/ataxia syndrome (FXTAS), and fragile X primary ovarian insufficiency (FXPOI). Evidence of increased risk for additional medical, psychiatric, and cognitive features and conditions is now known to exist for individuals with a premutation, although some features have been more thoroughly studied than others. This review highlights the literature on medical, reproductive, cognitive, and psychiatric features, primarily in females, that have been suggested to be associated with changes in the *FMR1* gene. Based on this review, each feature is evaluated with regard to the strength of evidence of association with the premutation. Areas of need for additional focused research and possible intervention strategies are suggested.

## Review

The fragile X mental retardation 1 (*FMR1*) gene was discovered in 1991, and named as such for its role as the causative gene for fragile X syndrome (FXS) [1-3]. Over the last two decades since its discovery, significant advances have been made in understanding the phenotypic expressions of mutated *FMR1* alleles. The primary mutation known to cause FXS results from an expansion of a cytosine-guanine-guanine (CGG) trinucleotide repeat sequence in the first exon and promoter of *FMR1.* The repeat sequence is located in the 5′ untranslated region of the *FMR1* mRNA, and thus is not translated and does not affect the sequence or structure of its encoded product, the Fragile X Mental Retardation Protein (FMRP). Normal alleles are considered to have between 5 and 44 CGG repeats, alleles with 45 to 54 repeats are intermediate or ‘gray zone’, 55 to 200 repeats constitute a ‘premutation’ and 200 or more repeats, a ‘full mutation’.

An individual with a full mutation is considered to have FXS, which results from hypermethylation of the promoter, silencing of the *FMR1* gene and subsequent decrease or absence of production of FMRP, which is necessary for healthy brain maturation. The phenotype of FXS includes intellectual disability (ID), hyperarousal, social difficulty, anxiety, aggression, and autism spectrum disorder (ASD) or autism traits [4]. The FXS phenotype is not described in detail here; however, literature on the impact of the challenging behavioral characteristics of FXS on family members with a premutation is included where applicable.

The premutation was historically thought to be associated only with risk for instability in the gene from generation to generation, and for children or grandchildren with FXS, and not to specifically mediate disease in the premutation ‘carriers’ themselves. However, research on the premutation phenotype over the last 10 to 15 years has demonstrated clear health risks associated with *FMR1* expansions in the premutation range. Two known disorders - fragile X-associated primary ovarian insufficiency (FXPOI) and fragile X-associated tremor/ataxia syndrome (FXTAS) have now been well documented [5,6]. These conditions are known to exist in a subset of individuals with a premutation, with specific associated symptoms and trajectories. Several additional medical, emotional and cognitive challenges have also been described as occurring at a greater frequency among individuals with a premutation than would be expected in the general population, although the association of the premutation with many of these conditions is less well established. Why some individuals with the premutation are unaffected and others have symptoms may relate to a number of factors, including the CGG repeat length, magnitude of elevation of toxic CGG repeat-containing *FMR1* mRNA. Also possibly implicated are antisense *FMR1* transcripts, low FMRP levels, (particularly in the upper premutation range), byproducts of aberrant translation of the repeat sequence including polyglycine-containing peptides, genomic changes in the rest of the genome, and environmental factors including toxins or other exposures that may be harmful to the brain and stress in families with fragile X associated disorders. There is a suggestion of a continuum of involvement with mild anxiety or shyness in some children or adults with the premutation to more severe psychiatric problems and as carriers age, appearance of neurological problems that may eventually result in FXTAS [6].

Understanding the phenotype associated with the premutation is important from a public health perspective, given its prevalence. In the three US population-based studies of the prevalence of CGG expansions, a higher prevalence was observed than previously reported (1 in 151 women and 1 in 468 men [7]; 1 in 209 women and 1 in 430 men [8]; 1 in 148 women and 1 in 290 men [9]. This prevalence translates into over 1 million premutation carriers in the United States. The world literature reveals considerable ethnic variability of prevalence of the premutation, ranging in males from 1 in 1,674 in Japan to 1 in 251 in Spain, and in females from 0 detected in 324 tested cases in Japan to 1 in 113 in Israel (reviewed in [7]).

This paper provides a review of features described in the literature as being more prevalent in individuals, especially females, with a premutation. We approach this review with a focus primarily on features seen outside of the known diagnoses of FXS, FXTAS, and FXPOI, in order to identify additional conditions that may be directly associated with an *FMR1* expansion; however, we compare the relative strength of evidence for each feature among those with and without FXTAS or FXPOI in the tables. As there is more literature describing these features in females, we focus primarily on women; however, where mixed gender studies have been conducted, evidence of these features in males is also described. Similarly, we focus on the more extensive literature on adults with a premutation with reference where appropriate to studies that have included children. Where evidence or the suggestion of a relationship with CGG repeat length has been found, this relationship has been noted in the tables.

Based on the review of the literature and expert nominations from direct clinical experience, we describe medical, cognitive, and psychiatric features in females and rate each with the following criteria: 1) ‘definitely related’ indicating clear evidence of an association in unbiased or minimally biased groups of carriers relative to well-matched controls in several independent studies; 2) ‘probably related’ suggesting strong evidence of an association but either only one study, some conflicting evidence or a need for studies examining the broader population (for example, those not clinically ascertained); 3) ‘possibly related’ indicating emerging or anecdotal evidence of an association with more extensive research needed; or 4) ‘not likely related’ suggesting strong evidence of an absence of an association through direct examination. These ratings are not meant to be definitive; rather to assist in guiding future targeted research.

### Medical features

#### Immune mediated disorders

A variety of medical problems besides FXTAS and FXPOI have been reported to occur more frequently in premutation carriers ascertained from clinical populations, as compared to noncarrier controls. Thyroid problems were found in 17.3% of non-FXTAS female carriers (not significantly different from controls), but in 50% of women with FXTAS, which was a significantly higher frequency than in age-matched controls [10]. A study of female carriers by Rodriquez-Revenga *et al*. [11] also found a significant increase in thyroid disease, as did results from a large national survey of female carriers [Wheeler, A.C, Bailey D.B., Raspa M., unpublished data]. Comparison between nonclinically referred premutation carriers and non-carriers ages 18 to 50 showed an increase to 10% from 5%, but this was not statistically significant [12]. Thus, this association may be important in older women. Irrespective, these problems may include either a history of Hashimoto’s thyroiditis leading to hypothyroidism or occasionally, Graves’ disease. These problems are immune-mediated disorders (IMD) and they are one of several such problems that have been reported in female carriers [13]. Winarni *et al*. [13] studied 344 adult female carriers ascertained from fragile X families and found that 44.7% suffered from an IMD compared to 27.8% of controls. The odds ratio of having an IMD was significantly elevated both in females with FXTAS (OR = 5.5) and without FXTAS (OR = 2.1) from this cohort compared to controls [13].

#### Fibromyalgia symptoms

In the study of IMD [13], autoimmune thyroid disorders were the most common problems overall (24.4%) followed by fibromyalgia in 10.2%. Both of these were significantly different compared to a limited number of controls (n = 72). However, two recent studies of *FMR1* mutations among women with fibromyalgia in Spain have reported conflicting findings, with one study of 353 women finding an increased rate of *FMR1* premutations, while a second study of 700 women did not find an association [14,15]. More research is needed in this area.

#### Hypertension

Hypertension was first noted by Coffey *et al*. [10] in a survey of 146 premutation female carriers ascertained from fragile X families in clinic. In a large national survey of women with a premutation, 15.1% of women reported a diagnosis of hypertension, which was significantly lower than national occurrence rates [Wheeler, A.C, Bailey D.B., Raspa M., unpublished data]. Similarly, hypertension was seen in 16.4% of women without FXTAS which was not different from age-matched controls (10%). However, hypertension was seen in 61% of females with FXTAS, a significantly higher frequency than 18%, observed in age-matched controls. Hypertension may be considered an autonomic problem associated with premutation-mediated toxicity related to FXTAS.

#### Migraines

Migraines are common among carriers and they occur in both males and females. In a single study of 315 carriers (203 females and 112 males) compared to 154 controls, a diagnosis of migraines was seen in 54.2% of female carriers, significantly different from female controls (25.3%) [16]. Smith, Seltzer, and Greenberg [17] reported that headaches (not specifically migraines) were significantly more common among premutation carrier mothers of children with FXS (26.9%) than matched controls (13.6%) who did not have children with disabilities. Of note, headaches were not significantly increased in carriers ascertained from the general population compared to controls in Seltzer *et al*. [7], so this may imply a relationship between migraines in families ascertained in clinic and family stress issues. Migraines can be associated with some syndromes resulting from mitochondrial dysfunction, which has been observed in cell lines from premutation carriers [18,19]. As with the general population, the onset of migraines is typically in teenage or young adult years and migraine prevalence decreases with age [16].

#### Neuropathy

Neuropathy was first reported in female premutation carriers in a series of five case reports of females with FXTAS, of whom four had signs of neuropathy on examination [20]. In a subsequent neuropathological case series [21] of eight women with the premutation with or without FXTAS, three were noted to have neuropathy. One of these women had chemotherapy with multiple neurotoxic agents, making it difficult to determine whether the neuropathy related to the premutation, and one had multiple sclerosis with likely central symptoms complicating assessment of nerve disease. One of the women, however, did have neuropathy with no obvious cause other than the premutation.

Several studies have examined neuropathic signs and symptoms in large groups of carriers relative to controls. Berry-Kravis *et al*. [22] found a significantly higher numerical neuropathy score based on examinations of 73 female premutation carriers relative to 32 age-matched control women, but this finding did not achieve statistical significance. The neuropathy score correlated with CGG repeat length after controlling for activation ratio, suggesting a relationship of neuropathic signs to the premutation. Neuropathy scores also correlated with the ataxia subscore on the FXTAS rating scale, suggesting that carriers beginning to have signs of FXTAS are more likely to have neuropathic signs than those without signs of FXTAS. Coffey *et al*. [10] studied 128 female carriers without FXTAS, 18 carriers with FXTAS and 69 controls, and found a significant rate of numbness and tingling and muscle pain in the extremities in both women with FXTAS and also in those without FXTAS compared to controls. However, neurological examination showed there was rarely sensory loss or evidence of neuropathy in women without FXTAS. Evidence of neuropathy upon examination was increased in carriers with FXTAS as compared to controls and to carriers without FXTAS. Carrier women without FXTAS also reported a history of intermittent tremor in 11%, significantly higher than the controls with 1.5% [10]. Therefore, symptoms of neurological problems by history are more common than what is documented by neurological examination, presumably because symptoms may be very intermittent early on and persistent findings only occur when the CNS changes are more significant. In Chonchaiya *et al*. [23], a structured questionnaire was administered to 110 female carriers who were daughters of a parent with FXTAS, 36 female carriers without a parent with FXTAS, and 43 controls. Neuropathic symptoms in the arms and legs were more frequently reported by carriers with and without parents with FXTAS than by controls. Two other studies found a significantly higher prevalence of neuropathic symptoms. Seltzer *et al*. [7], in a population based sample, reported that 29% of premutation carriers reported numbness (versus 13% of controls), and Smith *et al*. [17] found that 26.6% of premutation carrier mothers of full mutation children with FXS experienced muscle soreness (versus 16.6% of controls). Hunter *et al*. [12] did not find an increase in reporting of musculoskeletal symptoms by carrier women compared to controls, although they did not specifically ask about neuropathic symptoms. Based on the literature, it seems that neuropathy is clearly related to carrier status in female carriers but may not become manifest until symptoms of FXTAS also begin to emerge.

#### Vestibular issues

Vestibular complaints with a feeling of spinning, suddenly being unbalanced, and difficulty with ‘equilibrium’ are commonly reported complaints voiced by female premutation carriers. In Chonchaiya *et al*. [23] dizziness was reported more frequently by carriers with or without a parent with FXTAS than by noncarrier controls, and balance problems by carrier women with a parent with FXTAS more frequently than by carriers with no parent with FXTAS or controls. Smith *et al*. [17] reported significantly higher rates of dizziness in premutation carrier mothers of children with FXS (5.0%) than controls (1.3%), and this was also reported by Seltzer *et al*. [7] who found that dizziness and faintness were experienced by 18% of premutation carriers in a population-based sample, significantly higher than controls (4%). Recent quantitative studies using computerized dynamic posturography (CDP), a ‘gold standard’ balance assessment tool that has been shown to be highly sensitive, have shown impairments relative to control standards in a small group of female premutation carriers with the vestibular conditions of the sensory organization test and with the motor control and limits of stability test [24]. Vestibular deficits appeared to be present in carriers who did not meet clinical criteria for FXTAS, but were correlated with FXTAS motor rating scale scores and were more severe in those with FXTAS.

#### Other symptoms

In addition to the symptoms reported above, Smith *et al*. [17] reported significantly higher rates of backache and fatigue in premutation carrier mothers of full mutation children with FXS than in controls.

Table 1 summarizes nominations for medical/health features for women with a premutation with and without a diagnosis of FXTAS.

**Table 1:** Medical/health features examined in premutation carriers

**Medical/health features**	**Strength of evidence-those with FXTAS**	**Strength of evidence-asymptomatic of FXTAS**	**Strength of evidence - association with genetic markers**
Thyroid Disease	Probably related	Possibly related	Possible association with CGG repeat length
Hypertension	Possibly related	Possibly related	Possible association with CGG repeat length
Fibromyalgia symptoms	Probably related	Possibly related	Possible association with CGG repeat length
Migraine	Possibly related	Possibly related	Not found to be associated with CGG repeat length
Neuropathy	Definitely related	Probably related	Severity related to CGG repeat length after controlling for AR
Vestibular difficulties	Probably related	Probably related	Relationship not yet examined

FXTAS- fragile X-associated tremor/ataxia syndrome.

### Reproductive features

#### Ovarian insufficiency

Female premutation carriers are known to be at increased risk for Fragile X-associated Primary Ovarian Insufficiency (FXPOI). FXPOI encompasses premature ovarian failure, or cessation of menses prior to age 40, and other indicators of early ovarian aging or dysfunction [25]. On average, the age at menopause among premutation carriers is 5 years earlier than in the general population of women [5,7,26]. Smith *et al*. [17] also reported significantly higher rates of hot flashes or flushes in premutation carriers (15.4%) than controls (6.9%). CGG repeat size has been found to be associated with the risk of earlier menopause but in a non-linear manner, with the highest risk for those with mid-range repeats (approximately 70 to 100) [5,27-30].

Among women who are still cycling, hormonal changes associated with FXPOI are noted more often in women with the premutation compared with controls. These include decreased levels of anti-Müllerian hormone (AMH) and increased levels of follicle stimulating hormone (FSH), among others [5,26,29,31-33]. Other signs of early ovarian aging include irregular, shorter or skipped cycles and subfertility [27,32].

#### Fertility issues

The most immediate and significant consequence of POI is reduced fertility [27,34]. Given the higher rates of early menopause, difficulty with fertility is significant for women with a premutation. Even among younger women, difficulty getting pregnant has been reported at higher than expected rates among women who are premutation carriers [27]. In a large national survey study of families affected by FX, female premutation carriers reported a significantly higher rate of use of assistive reproductive technology (for example, fertility drugs, IVF) *before knowing their FMR1 status* than is seen in the national occurrence rates [Wheeler, A.C, Bailey D.B., Raspa M., unpublished data]. Similar to the other symptoms of FXPOI, fertility problems appear to be most significant for women in the mid-range CGG group [27].

#### Obstetric and perinatal difficulties

Despite known challenges with fertility, very little is known regarding potential obstetric or perinatal risks for *FMR1* premutation carriers. One study, conducted in Finland, examined pregnancy outcomes in 63 women who were premutation carriers compared to the general obstetric population [35]. The authors of this study found a slightly higher risk for late pregnancy bleeding in the *FMR1* premutation, but no other concerns related to the course or outcome of pregnancy. In a large national survey study [Wheeler, A.C, Bailey D.B., Raspa M., unpublished data] a significantly higher rate of reported preeclampsia was found in premutation carrier women than would be expected based on national occurrence rates in the United States. More research is needed to determine whether women with a premutation experience greater pregnancy or birth risks.

#### Estrogen-deficiency related conditions

In general, the state of early estrogen deficiency resulting from POI leads to an increased risk for low bone density, earlier onset osteoporosis and bone fractures [36], impaired endothelial function [37], earlier onset of coronary heart disease [38], and increased cardiovascular mortality. Among premutation carriers, lower bone mineral density [39] and osteoporosis [27] is reported at a high frequency than among non-carriers, but not other estrogen deficiency related disorders.

Hypoestrogenism may have behavioral and cognitive consequences as well. Women with an earlier age at menopause are reported to have more anxiety, depression, somatization, sensitivity, hostility, and psychological distress than women with normal ovarian function [40]. Some studies have found suggestive evidence that symptoms of FXPOI may partially explain the increased vulnerability for mood disorders [41] and anxiety and depression [12].

Given the altered hormone profiles and the stress related to being a carrier of the premutation (for example, additional potential stress of having a newborn with FXS), postpartum depression would seem to be of great concern. Very few studies have specifically examined increased risks for PDD in women with a premutation. Wheeler *et al*. [unpublished data] found that rates of self-reported postpartum depression among women with a premutation were not higher than is found in national occurrence rates. However, among those who had experienced PPD, 41.18% experienced at least 2 episodes. In a study of 50 women with a premutation who also had children and a history of major depressive disorder, there was a significant increase in risk for PDD among women with more than one child with FXS [42]. Therefore it may be the cumulative stress of having multiple children with FXS that increases risk for PDD, rather than premutation status. More research is needed in this area.

The question of whether or not endogenous or exogenous estrogen (from replacement therapy) is related to indicators of neurocognitive deficit is unresolved (reviewed in [43]). The statistically significant association of reduced Verbal IQ and a crude measure of estrogen status among premutation carriers suggests further studies should be done [44].

Estrogen also plays a role in immune-response and inflammation and has been implicated in the onset of autoimmune disorders [45], a class of disorders reported to be elevated among women who carry the premutation carries as noted above. Lastly, estrogen has been shown to moderate the impact of chronic stress on mood and psychiatric outcomes [46] a role of particular interest given that women with the premutation often experience high levels of stress related to their role as primary caretakers of children with fragile X syndrome and older relatives with FXTAS.

Table 2 summarizes nominations for reproductive features for women with a premutation with and without probable FXPOI.

**Table 2:** Reproductive features examined in female premutation carriers with and without FXPOI

**Reproductive features**	**Strength of evidence - those with FXPOI**	**Strength of evidence-those without FXPOI**	**Strength of evidence - association with genetic markers**
Ovarian insufficiency	Definitely related	Definitely related	Strong evidence of nonlinear associate with CGG
Estrogen-related conditions	Definitely related	Possibly related	Possible association with CGG repeat length
Fertility issues	Definitely related	Definitely related	Strong evidence of nonlinear associate with CGG
Obstetric and perinatal difficulties	Possibly related	Possibly related	Relationship not yet examined
Postpartum depression	Possibly related	Possibly related	Relationship not yet examined

FXPOI- fragile X-associated primary ovarian insufficiency.

### Neurocognitive features

Various cognitive domains have been reported to be affected by the premutation even in those without FXTAS. These domains include executive function [47,48], working memory [49], and arithmetic [50]. These deficits can appear even in young individuals and often show a more progressive course in premutation carriers than in the general population. This may be an early sign of the often significant cognitive impairment, primarily in the realm of executive dysfunction that can accompany FXTAS [51]. However, it must be noted that some studies have not found these areas of weakness. Most likely there is a subset of women with the premutation who are more vulnerable to effects of the premutation.

#### General intelligence

Several studies have found normal overall cognitive abilities in non-FXTAS female adult premutation carriers who underwent general intelligence tests (most commonly the Wechsler scales) [47,52-56]. A recent study in older female premutation carriers over age 50 also showed normal IQ scores [57]. In contrast, a few reports have documented lower verbal IQ scores among these women compared to female normal controls [44,58] or their male counterparts [59], with CGG repeat length explaining approximately 4% of the variance of verbal IQ per linear regression [44]. Case studies have shown mixed results as well, with low [60,61] and superior [62] IQ scores both observed in girls with the premutation. Myers and colleagues [63] examined 14 children (7 female) and found a trend towards lower performance IQ, a measure closely related to executive functioning. Arithmetic difficulties have also been reported in females with the premutation [50,58].

#### Executive function

Executive function involves the capacity for self-regulation of behavior and attention, and consists of multiple processes including maintaining and updating relevant information in working memory, inhibition of irrelevant information, switching task goals, and performance monitoring [64,65]. Although executive function has been regarded as the primary cognitive domain affected in patients with FXTAS [50,66], several studies of non-FXTAS female premutation carriers in young adulthood and midlife have reported normal scores on widely-used executive functioning tests such as the Wisconsin Card Sorting Test (WCST), Trail Making Test (TMT) and Stroop Color and Word Test [52,54,55,67]. Whereas, in a study involving older female premutation carriers up to 76 years of age (mean age = 41) [47], executive function, as measured by the Behavioral Dyscontrol Scale (BDS) [68], was modulated by premutation CGG repeat length and/or FMRP level after controlling for full-scale IQ scores (FSIQ). By examining event-related brain potentials (ERPs) in older female premutation carriers, Yang *et al*. [57] found poorer BDS performance correlated with decreased amplitude of the frontal P300. This ERP component is thought to index selective attention and working memory updating processes, which may underlie the executive dysfunction in both males and females with FXTAS [57,69]. Thus, executive dysfunction likely presents as a late-onset phenotype affecting non-FXTAS female premutation carriers over age 50 in addition to non-FXTAS premutation males [48,66]. It is also worth noting that both of the two studies [47,57] with findings of executive dysfunction have controlled for FSIQ as a covariate.

#### Attention

Sustained attention in a visual search task was comparable among non-FXTAS females with the premutation and various control groups [58]. A large sample of females with the premutation had more self-reported attention deficits than controls without a mutation [55]. Attention involved in enumerating 5 to 8 rectangles was shown to be modulated by both premutation CGG repeat length and age [70].

#### Memory

Verbal memory generally remains intact in non-FXTAS female premutation carriers [55,57,58,67], but one case study reported substantially impaired verbal memory functions in a 72-year-old old woman [71]. Both immediate and delayed recall of visual memory has been shown to be affected by the premutation CGG repeat length in these carriers [47]. In a functional magnetic resonance imaging (MRI) experiment using a working memory task, carriers of both genders showed reduced frontal activation regardless of FXTAS diagnosis [72]. Interestingly, Yang *et al*. [57] found impairments in some measures of working memory in non-FXTAS female carriers that were not present in females with FXTAS.

#### Language issues

As noted earlier, studies assessing global indices of verbal ability such as verbal IQ, have generally reported that premutation carriers who are not affected by FXTAS exhibit comparable abilities as matched controls or normed references (reviewed in [12]). In contrast, results from the few studies that have assessed functional language (or, the use of language in social contexts, for example, conversational ability) suggest that such language skills may be impacted among premutation carriers. Sterling *et al*. [73] elicited brief language samples collected from a group of nearly 200 women with the premutation and assessed samples for different types of dysfluencies, including incomplete or abandoned utterances, repetitive speech, excessive fillers (for example, um, ah, oh), and revisions to correct or modify a prior utterance. These types of dysfluencies could reflect problems with executive skills such as planning and organization, and can interfere with fluent communication. As such, they may represent clinically meaningful phenotypes. In this study, the rate and types of dysfluencies were compared between women with the premutation and women who were parents of individuals with autism, included to control for the stress of caring for a child with a disability, which the authors hypothesized could impact fluency. Analyses revealed significantly higher rates of all types of dysfluencies among the premutation carrier group (compared to the mothers of children with autism), as well as a significant association with age (increased dysfluencies with greater age). There was no association with CGG repeat length, and other molecular variables were not examined. Losh *et al*. [74] also reported elevated rates of subtle conversational differences among premutation carrier women, further suggesting that language may be affected in the premutation.

Table 3 summarizes nominations for cognitive features in females with a premutation with and without a diagnosis of FXTAS.

**Table 3:** Cognitive features examined in female premutation carriers with and without FXTAS

**Cognitive features**	**Strength of evidence- those with FXTAS**	**Strength of evidence-asymptomatic of FXTAS**	**Strength of evidence - association with genetic markers**
Low IQ	Definitely related	Probably related	Possible association with CGG repeat length
Arithmetic difficulties	Definitely related	Probably related	Possible association with CGG repeat length
Executive dysfunction	Definitely related	Probably related	Probable association with CGG repeat length
Attention deficits	Definitely related	Possibly related	Probable association with CGG repeat length
Verbal memory	Definitely related	Not likely related	Possible association with CGG repeat length
Verbal working memory	Definitely related	Probably related	Possible association with CGG repeat length
Visual memory	Definitely related	Probably related	Possible association with CGG repeat length
Language	Definitely related	Probably related	Not found to be associated with CGG repeat length

FXTAS- fragile X-associated tremor/ataxia syndrome.

### Psychiatric features

#### Affective disorders

Mood disorders have been a concern in the fragile X premutation population for over two decades. Initial small studies in women failed to demonstrate meaningful difference between carriers and controls in depressive disorders [52,75]. However, these studies were small, limited by the screening tools and by poor recall. Later, much larger research studies did demonstrate a relationship between the number of CGG repeats and the presence of depression [76] as well as the severity of depressive symptoms [77]. When comparing 93 women with the premutation screened using the strict criteria of the structured clinical interview for the DSM-IV (SCID) to a large national databank, the lifetime prevalence of major depressive disorder was 43.0% versus 31.9% [50]. Although Seltzer *et al*. [7] did not find evidence of elevated prevalence of depressive symptoms in a population-based sample, and Smith *et al*. [17] similarly did not report elevated rates of negative affect in premutation carriers than controls. Seltzer *et al*. [78] reported evidence of higher rates of depressive and anxiety symptoms in premutation carrier mothers of full mutation children with FXS under certain genetic and environmental conditions: mothers who experienced stressful life events in the previous year had higher rates of depression and anxiety if their CGG repeats were in the mid-range of CGG repeats. Dysthymia and bipolar disorder have generally failed to demonstrate significant levels in carriers compared to controls [79].

#### Autism spectrum disorders

Because FXS is one of the most common single-gene disorders associated with autism, investigation has been undertaken to determine what risk the carrier state confers. In a screening study of individuals from families with FXS, roughly 14% of boys and 5% of girls with the premutation were found to also have an autism spectrum disorder (ASD) [80]. Even among those carriers not diagnosed with ASD, related psychological traits are more common among carriers compared to controls without the premutation. A relatively large body of research has now documented differences among parents and relatives of individuals with autism, constituting a broad autism phenotype, or, a constellation of mild behavioral features that resemble the features of autism in quality but that are more subtly expressed and not typically associated with functional impairment (reviewed in [81]). A recent study examined a broad range of pragmatic language skills as well as related behavioral features of the broad autism phenotype among women with the premutation, compared with mothers of children with autism, and mothers of typically developing children with no family history of fragile X, autism, or language impairment [74]. In this study, conversational samples from a videotaped semistructured interview were used to assess pragmatic language using the Pragmatic Rating Scale (PRS) [82]. This study replicated previous findings in the autism parent group and also showed that women with the premutation exhibited similarly elevated rates of pragmatic language problems relative to controls. Factor analysis of items on the PRS revealed that premutation carriers committed the same types of pragmatic language violations as parents of individuals with autism. Such similarities in pragmatic language profiles could suggest that the differences between these groups stem from similar underlying factors, supporting a link between *FMR1* and autism-related phenotypes in carrier relatives. In support of this possibility, the study also found that premutation carriers displayed elevated rates of personality traits associated with the broad autism phenotype, rigid or inflexible traits and socially reticent dispositions. The presence of broad autism phenotype traits was associated with greater expression of autism symptoms in their children with FXS. Other studies have also found increased rates of both social aloofness [83] and a rigid perfectionism [84] among carrier women. Studies have shown that amygdala dysfunction in viewing social stimuli seen in carriers correlates with clinical findings of social deficits and both FMRP deficits and elevated *FMR1* mRNA levels [85].

#### Anxiety

The largest and most recent study of life-time mood and anxiety in the premutation population was completed by Bourgeois *et al*. [79]. In that study, carriers both suffering and not suffering from FXTAS were compared in the prevalence of anxiety disorders to a very large age-matched national dataset. In terms of all anxiety disorders, only those suffering from FXTAS demonstrated a higher prevalence. When separated out, this was similarly true for panic disorder, post-traumatic stress disorder and specific phobia. Generalized anxiety disorder and obsessive compulsive disorder failed to demonstrate any difference between carriers and controls. Only social phobia was found to have higher levels in premutation carriers without FXTAS compared to controls. Chronic anxiety has also been associated with radiological signs on MRI; specifically, the higher the anxiety score the smaller the size of the hippocampus in women with the premutation [86].

Other reports have not found high levels of mental health problems; however, differences in measurements (life-time versus state traits, interview versus self-report), age of participants, ascertainment of participants, *etcetera*, will all play a role in explaining such differences. Irrespective, continued research to identify vulnerable women is essential.

#### Psychosis

Psychosis, given its relative rarity in the general population, has been challenging to study in premutation carriers. Initial linkage analysis failed to show a clear relationship of schizophrenia to the *FMR1* gene [87]. Prevalence studies have found the overall rate of psychotic disorders to be low [83]. There have, however, been several case reports of combined psychotic illnesses and the premutation, including schizoaffective disorder [88] and a subject with combined schizophrenia and schizoid personality disorder [89]. Interestingly, as opposed to frank psychotic disorders, multiple studies have found increased prevalence of schizotypal personality traits in the carrier population [83,90].

#### Attention deficit/hyperactivity disorder

Attention regulation difficulties have been proposed to be a problem in people with the premutation. Notably, when compared with their control siblings, premutation carriers had significantly more issues with attention than their noncarrier siblings [91]. Inattention and impulsivity amongst *FMR1* carriers can be problematic through adulthood [55], although hyperactivity was not noted to be increased in prevalence. In an analysis to examine the genetic architecture of ADHD symptoms in families with FXS, it was found that the *FMR1* repeat accounts for about 5% of the variance whereas polygenes account for about 50% of the residual variance. This suggests that the premutation acts in concert with additional genetic loci to influence the severity of ADHD symptoms [92].

#### Sleep and related problems

Sleep issues have recently been studied in carriers, and common measures such as the Pittsburgh Sleep Quality Index and the Insomnia Severity Index have shown significant pathology. These findings may be related to higher rates of Restless Legs Syndrome and sleep apnea, both of which have shown multiple-fold increases in subsets of the premutation population [93,94]. Sleep apnea is common in older carriers with FXTAS. In a study of 229 males and 201 females with the premutation, including 118 with FXTAS and 123 controls, sleep apnea occurred in 31.4% of carriers with FXTAS, 8.6% of carriers without FXTAS and in 13.8% of controls by medical history [93]. These issues may be contributing to the development of significant fatigue, which is a common complaint of adult carriers [95].

#### Stress susceptibility

Two hypotheses have been advanced to account for elevated psychiatric, cognitive, and somatic symptoms in premutation carriers [96]: first, that such symptoms are a primary biological feature of *FMR1* CGG expansions in the premutation range, and second, that such symptoms may be exacerbated by the stress associated with parenting a child with FXS. Importantly, stress may interact with the biological vulnerabilities caused by the premutation, and thus it is important to incorporate biological markers when exploring stress effects in this population.

##### Parenting stress

It has been well established that many individuals with FXS have severe behavior problems, including inattention, hyperactivity, aggression, anxiety, and autism symptoms [97], which result in high levels of stress exposure for their families [96,98-102]. In one study, exposure to child behavior problems was investigated in the context of a biomarker of the premutation, namely the activation ratio. Hartley *et al*. [103] studied the prevalence of daily behavior problems in adolescents and adults with FXS who were living with their families, and found that during an 8-day period, 85.7% exhibited at least one episode of significant behavior problems, such as behaviors that were disruptive, aggressive, destructive, self-injurious, unusual or repetitive, socially offensive, uncooperative, or inattentive. Such episodes occurred, on average, about every other day.

The effects of this level of stress exposure were evident in the mother’s level of cortisol, a stress hormone that previously has been shown to be depressed in mothers of children with developmental disabilities, including autism [104,105]. Hartley *et al*. [103] found that premutation carrier mothers who had a low activation ratio (AR; that is, a low proportion of cells expressing the normal allele) showed an atypical response to their child’s behavior problems. Specifically, for mothers with a low AR, the greater the number of behavior problems manifested by their child the previous day, the lower the mother’s cortisol level the next morning, which is an atypical response to acute stress. In contrast, for mothers with a high AR (that is, a larger proportion of cells expressing the normal allele), the greater the number of child behavior problems the previous day, the higher the mother’s morning cortisol, which is a more normative neuroendocrine response to environmental stress. Also, mothers who had more than one child with FXS had lower cortisol levels than those who had ‘only’ one affected child, further supporting the toll that stressful parenting takes on neuroendocrine functioning in carrier mothers.

Hunter *et al*. [106] hypothesized that among women with the premutation, the stress of raising a child with FXS may be moderated by genetic factors influencing endogenous cortisol responses, which could in turn modulate mental health symptoms. They examined the association of genetic variation in the corticotrophin releasing hormone receptor 1 locus (*CRHR1*) in 460 women, including premutation carriers with and those without a child with FXS and non-carriers. A statistically significant interaction between the *CRHR1* genotype and the status of raising a child with FXS was associated with social anxiety symptoms as reported on the Social Phobia and Anxiety Inventory (SPAI). These preliminary data suggest that there may be a subgroup of premutation carriers who are more susceptible to the effects of stress.

##### Stressful life events

Stressful life events are a source of stress quite distinct from child behavior problems, and include experiences such as divorce, death of a family member or close friend, caring for an aging parent, or negative changes in financial or health status. These life events have been shown to compromise psychological well-being in the general population [107] and in parents of children with developmental disabilities [108,109]. Although exposure to child-related stress is a common characteristic of premutation carrier mothers of children with FXS, these mothers vary with respect to their exposure to other types of stress.

Only one study has been conducted investigating general life stress effects in premutation carrier mothers of children with FXS. Seltzer *et al*. [78] found that during the previous year, two-thirds (68.3%) reported having experienced at least one negative life event. However, the other mothers did not experience any of these sources of life stress during the previous year. The variation in exposure to life events made it possible to investigate the effect of exposure to this type of stress on premutation carrier mothers of children with FXS.

Specifically, Seltzer *et al*. [88] investigated the effects of exposure to stressful life events with respect to depressive and anxiety symptoms as well as cortisol level in premutation carrier mothers. They found that the higher number of stressful life events experienced during the previous year, the greater the level of depression and anxiety, and the lower the level of cortisol. However, variation in the biology of the premutation was critical to understanding the pattern of stress effects. A curvilinear association was observed between stress exposure and CGG repeat length. Premutation carrier mothers with mid-range CGG repeat lengths (approximately 90 to 105) had the highest levels of depression, anxiety, and the most abnormal cortisol parameters if they had recently experienced stressful life events, but mothers in the same CGG repeat range had the *lowest* level of depression and anxiety, and the most normal cortisol parameters if their life had been free from stressful events in the previous year. This divergence was most prominent in the mid-CGG range, whereas those with low CGG repeat numbers or repeats closer to the full mutation cut-off were less reactive to stress. A curvilinear pattern of vulnerability in CGG repeat effects has been previously shown for reproductive outcomes in carrier females [5,26,27] and for depression [56]. This study implicates exposure to stressful life events and CGG repeat length in the manifestation of psychiatric symptoms in premutation carrier mothers.

A large number of studies have shown that autism is an extremely stressful developmental disorder, arguably the disorder posing the greatest level of parenting stress [110,111]. A few studies have contrasted parenting stress in mothers who have a child with autism and mothers whose child has FXS [17,112]. These studies generally have found similar patterns for premutation carrier mothers of children with FXS and mothers of those with autism. These findings of similarity between premutation carrier mothers of children with FXS and mothers of children with autism are similar to earlier work in which it was found that the most consistent predictor of maternal depression across disability groups (FXS, ASD, and Down syndrome) was child behavior problems [113]. Only when research that separates the effects of the biology of *FMR1* expansions from the effects of stressful parenting will there be a complete understanding of the premutation phenotype and how stress may alter it.

Table 4 summarizes nominations for psychiatric features in premutation carriers.

**Table 4:** Psychiatric features examined in female premutation carriers with and without FXTAS

**Psychiatric features**	**Strength of evidence- those with FXTAS**	**Strength of evidence-asymptomatic of FXTAS**	**Strength of evidence - association with genetic markers**
Affective disorders	Probably related	Probably related	Possible association with mRNA, CGG repeat length
Anxiety	Possibly related	Possibly related	Possible association with mRNA, CGG repeat length
Autism traits	Not examined	Possibly related	Relationship not yet examined
Psychosis	Possibly related	Possibly related	Possible association with mRNA, CGG repeat length
ADHD	Possible related	Probably related	Possible association with mRNA, CGG repeat length
Sleep issues	Possibly related	Possibly related	Relationship not yet examined

FXTAS- fragile X-associated tremor/ataxia syndrome.

## Conclusions

At this point there is strong evidence to suggest that females with an *FMR1* premutation may be variably at risk for multiple medical, reproductive, cognitive, and psychiatric difficulties. While there are clear phenotypes associated with specific *FMR1-*related diagnoses (FXS, FXTAS, FXPOI), there are many more diagnoses that may be associated with the premutation and should be considered whenever a premutation carrier presents to a clinic. Several recent papers have described significance and breadth of concerning features reported to be associated with the *FMR1* premutation [6,11,114,115], thus capturing the importance of better describing phenotypes associated with the premutation.

Based on this review of the literature, we have identified specific features thought to be at higher risk for premutation carriers as being ‘definitely related’ ‘probably related’ ‘possibly related’ or ‘not likely related’ to the molecular changes associated with an *FMR1* expansion. While the rates of many medical and psychiatric problems are significantly increased in aging carriers who have FXTAS [79], the onset of some of these problems has been reported well before the onset of an official diagnosis [116]. The emergence of some problems, such as ADHD, anxiety disorders and autism traits are noted to occur in some premutation carriers in childhood [91,117,118]. However, little is known about the early developmental phenotype of individuals with a premutation, and those studies that have focused on features in children with a premutation are virtually all based on clinically referred children or siblings of an individual with FXS. Additional longitudinal studies are needed to determine at what point some of these features may develop, whether they are developmental or degenerative and what protective factors might reduce risks for more negative outcomes.

CGG repeat length has been implicated in the onset and severity of several of these features. This is most notable in the literature on FXPOI, with a nonlinear association (greater severity among those with midrange repeats) being reported in multiple studies [5,27,28,30]. Similar nonlinear findings have been reported for psychiatric [12,56,78,119] symptoms, while other studies report linear associations between repeat length and severity of neurological symptoms [22,49,120]. There may be a variable degree of RNA toxicity in carriers due to elevation of the *FMR1* mRNA, which can lead to oxidative stress and neuronal hyperexcitability [121]. *FMR1* mRNA level increases with the length of the CGG repeat, and the expanded repeat-containing mRNA makes hairpin loop structures that appear to sequester important proteins for neuronal function including Sam 68, DROSHA and DGCR8 [122,123]. These later two proteins are necessary for maturing the miRNA, which regulate both transcription and translation in the CNS [123]. The RNA toxicity is thought to be related to miRNA dysregulation which can jeopardize survival of the neuron and glia containing the premutation [124,125]. Additional research is needed to examine the relationship between these biomarkers and phenotypic features.

Also, cumulative effects of multiple risk factors may occur. Those individuals with the premutation and either intellectual disabilities, seizures or autism traits are also likely to have a second genetic hit [R Lozano, RH, and F Tassone, unpublished data]. Hypertension and hypothyroidism, irrespective of the strength of the association with the premutation, should be evaluated medically and if present, treated, since the lack of treatment may aggravate CNS dysfunction. Neurological problems including migraines, neuropathy, sleep apnea and psychiatric problems should be considered and if present and sufficiently symptomatic then treatment should be initiated. Exercise, stress reduction techniques such as therapy, biofeedback or meditation, avoidance of toxins, such as excessive alcohol use or illicit drugs, avoidance of vitamin deficiency and healthy eating should be recommended for all carriers [6].

It is important to note that almost all research describing the premutation carrier phenotype is based on individuals who were ‘reverse-ascertained’ from a child with the full mutation in a clinical setting, and this potentially confounds the effects of the premutation with the impact of stressful parenting and referral bias. Thus, the literature may be skewed toward larger repeats, and more serious symptoms, as well as substantial ascertainment bias toward patients and families who are more likely to seek medical care for themselves or their child with FXS, and thus are more likely to have clinical symptoms. Studies of premutation carriers who do not have children with FXS and even who are not aware of their genetic status would make it possible to address a central unanswered question namely whether, in an unbiased sample of individuals with *FMR1* CGG expansions (who, for example, are not exposed to stressful parenting, and are not aware of literature related to their genetic status), there are increased risks of clinical or sub-clinical symptoms (neurocognitive, health, and psychiatric) and whether the severity of such symptoms is associated with their *FMR1* genotype.

## Abbreviations

ASD: autism spectrum disorder; BDS: behavioral dyscontrol scale; CDP: computerized dynamic posturography; ERPs: event-related brain potentials; FSIQ: full-scale IQ scores; FXPOI: fragile X-associated primary ovarian insufficiency; FXS: fragile X syndrome; FXTAS: fragile X-associated tremor/ataxia syndrome; ID: intellectual disability; IMD: immune-mediated disorders; MRI: magnetic resonance imaging; PRS: pragmatic rating scale; SCID: structured clinical interview for the DSM-IV.

## Competing interests

Bailey has received funding from Novartis Pharmaceuticals. Hagerman has received funding from Novartis, Roche, Seaside Therapeutics, Curemark, and Forest for treatment studies in fragile X syndrome or autism. Summers has received funding for research from Seaside Therapeutics, Roche, Novaris, and Marinus Pharmaceuticals.

## Authors’ contributions

AW drafted the introduction and conclusion and organized contributions from all authors; EBK contributed sections on neuropathy and vestibular issues; JG, MM, and LS contributed sections on stress responsivity; ML contributed the language and autism sections; MM, SSherman, and LRR contributed the reproductive section; SSummers contributed the psychiatric section; JO and JCY contributed the cognitive section; RH contributed to the medical section as well as to the introduction and conclusion. All authors read and approved the final manuscript.
